# Cryptococcal Antigen Screening in Patients Initiating ART in South Africa: A Prospective Cohort Study

**DOI:** 10.1093/cid/civ936

**Published:** 2015-11-12

**Authors:** Nicky Longley, Joseph Nicholas Jarvis, Graeme Meintjes, Andrew Boulle, Anna Cross, Nicola Kelly, Nelesh P. Govender, Linda-Gail Bekker, Robin Wood, Thomas S. Harrison

**Affiliations:** 1 Desmond Tutu HIV Centre, Institute of Infectious Disease and Molecular Medicine, University of Cape Town, South Africa; 2 Institute for Infection and Immunity, St. George's University of London, United Kingdom; 3 Department of Medicine and Institute of Infectious Disease and Molecular Medicine, University of Cape Town, South Africa; 4 Botswana-Upenn Partnership, Gaborone; 5 Division of Infectious Diseases, Department of Medicine, Perelman School of Medicine, University of Pennsylvania, Philadelphia; 6 Department of Clinical Research, Faculty of Infectious and Tropical Diseases, London School of Hygiene and Tropical Medicine, United Kingdom; 7 School of Public Health and Family Medicine and Institute of Infectious Disease and Molecular Medicine, University of Cape Town; 8 Health Impact Assessment Directorate, Department of Health, Provincial Government of the Western Cape; 9 National Institute for Communicable Diseases, a Division of the National Health Laboratory Service–Centre for Opportunistic, Tropical and Hospital Infections; 10 Faculty of Health Sciences, University of the Witwatersrand, Johannesburg, South Africa

**Keywords:** HIV, cryptococcal meningitis, cryptococcal antigen, antiretroviral therapy, screening

## Abstract

***Background.*** Retrospective data suggest that cryptococcal antigen (CrAg) screening in patients with late-stage human immunodeficiency virus (HIV) initiating antiretroviral therapy (ART) may reduce cryptococcal disease and deaths. Prospective data are limited.

***Methods.*** CrAg was measured using lateral flow assays (LFA) and latex agglutination (LA) tests in 645 HIV-positive, ART-naive patients with CD4 counts ≤100 cells/µL in Cape Town, South Africa. CrAg-positive patients were offered lumbar puncture (LP) and treated with antifungals. Patients were started on ART between 2 and 4 weeks and followed up for 1 year.

***Results.*** A total of 4.3% (28/645) of patients were CrAg positive in serum and plasma with LFA. These included 16 also positive by urine LFA (2.5% of total screened) and 7 by serum LA (1.1% of total). In 4 of 10 LFA-positive cases agreeing to LP, the cerebrospinal fluid (CSF) CrAg LFA was positive. A positive CSF CrAg was associated with higher screening plasma/serum LFA titers.

Among the 28 CrAg-positive patients, mortality was 14.3% at 10 weeks and 25% at 12 months. Only 1 CrAg-positive patient, who defaulted from care, died from cryptococcal meningitis (CM). Mortality in CrAg-negative patients was 11.5% at 1 year. Only 2 possible CM cases were identified in CrAg-negative patients.

***Conclusions.*** CrAg screening of individuals initiating ART and preemptive fluconazole treatment of CrAg-positive patients resulted in markedly fewer cases of CM compared with historic unscreened cohorts. Studies are needed to refine management of CrAg-positive patients who have high mortality that does not appear to be wholly attributable to cryptococcal disease.

## Abstract (precis)

Treating cryptococcal antigen (CrAg)-positive, antiretroviral therapy naiive patients with preemptive fluconazole resulted in markedly fewer cases of cryptococcal meningitis compared with unscreened historic cohorts. However, the same CrAg-positive patients experienced excess mortality not directly attributable to cryptococcal disease.

**(See the Editorial Commentary by Jackson and Horst on pages 588–9.)**

Cryptococcal meningitis (CM) is a leading cause of death in late-stage human immunodeficiency virus (HIV)–infected patients in much of the developing world [1–5]. In many low-resource settings, patients with advanced immunosuppression continue to present late to antiretroviral therapy (ART) treatment programs, and many die of HIV-related illness in the weeks just prior to and months following initiation of ART. CM causes many of these deaths and is also a heavy burden on healthcare facilities [6–11]. CM treatment remains inadequate, with an acute mortality ranging from 25% to more than 50% with current antifungal therapies [12].

Many of these CM cases may be preventable. Retrospective data have shown that routine screening for cryptococcal antigen (CrAg) to detect subclinical disease in patients presenting to ART programs can identify which patients are at risk of developing CM [13, 14]. Once identified, these patients could then be given preemptive treatment with high-dose fluconazole to prevent them from developing severe disease [15]. This strategy is likely to be cost-effective [16–18] and has been endorsed in World Health Organization guidelines [19] and adopted by some African countries [20].

However, despite early programmatic rollout, prospective data on the implementation and efficacy of screening are limited, as are data to guide how best to manage patients who test antigen positive. We undertook a prospective study of screening for CrAg in the plasma, serum, and urine of patients with late-stage HIV infection who enrolled for ART at 2 clinics in Cape Town, South Africa.

## METHODS

### Participants and Procedures

Consecutive, HIV-positive, ART-naive patients with no prior history of cryptococcal disease, aged >18 years, and with a CD4 cell count ≤100 cells/µL presenting to 2 ART clinics in Cape Town between May 2011 and April 2014 were approached for enrollment. Consenting patients were screened for CrAg with the lateral flow assay (LFA; IMMY, Norman, Oklahoma) using serum, plasma, and urine samples. The final 192 patients recruited also had an LFA performed on whole blood. At completion of recruitment, we determined CrAg titers on stored serum, plasma, and urine samples from patients who screened positive for CrAg using the CrAg LFA-IMMY kit, as per manufacturer's instructions. Serum was also tested using the latex agglutination assay (LA; CALAS system, Meridian Biosciences, Cincinnati, Ohio).

At the start of the study, the LFA was not approved (neither conformite Europeenne marked nor US Food and Drug Administration approved), and the initial 150 patients screened were treated on the basis of the LA test result; the test had also been used to demonstrate that screening identifies those at risk, and not at risk, of clinical cryptococcal disease [7]. During this period, LA-negative but LFA-positive patients received no antifungal therapy. As soon as it became clear that a significant number of patients were positive by LFA but not by LA, a protocol amendment was made to enable antifungal therapy to be given to patients who tested positive by either test.

Patients positive for CrAg were questioned and examined for symptoms and signs of meningitis. They had blood cultures performed and were offered a lumbar puncture (LP) to exclude early subclinical CM. Patients with evidence of meningeal involvement on the basis of positive cerebrospinal fluid (CSF) CrAg-LFA were admitted for intravenous amphotericin followed by fluconazole, as per local guidelines [12]. Those with negative CSF CrAg or declining LP were started on oral fluconazole: 800 mg daily for 2 weeks, 400 mg daily for 8 weeks, and 200 mg daily thereafter. All patients were started on efavirenz-based ART 2–4 weeks after CrAg screening, as per local guidelines. Patients on rifampicin had the dose of fluconazole increased by 50%.

Patients who screened CrAg positive were followed up every 2 weeks for 10 weeks and every 3 months thereafter for the first year of ART. Those screening CrAg negative were seen by clinic staff, and prospective data were collected every 3 months for the first year of ART. Telephone follow-up and home visits were undertaken for missed visits. Follow-up data were also collected using the National Health Laboratory Service electronic database, the Department of Home Affairs death register, and the provincial government electronic database systems. CrAg-negative patients were cross-referenced with the National Institute for Communicable Diseases' national surveillance GERMS South Africa database (http://www.nicd.ac.za/?page=germs-sa&id=97) to detect cases of CM diagnosed outside the Western Cape province .

The research ethics committees of the University of Cape Town and St. George's University of London approved the study. All participants gave written informed consent.

### Outcomes

The primary clinical outcome measure was incidence of CM in the first year of ART in all screened patients. Secondary outcomes were time to ART in CrAg-positive and CrAg-negative patients, the proportion of CrAg-positive patients who developed cryptococcal immune reconstitution inflammatory syndrome, and all-cause mortality.

The primary laboratory outcome measure was the prevalence of cryptococcal antigenemia. Secondary outcomes were the concordance of the serum LA CrAg test; the serum, plasma, whole blood, and urine LFA CrAg tests; and the relationship of antigen titer to CSF findings.

### Statistical Analyses

Data were analyzed using Stata (v. 13, StataCorp). Variables were compared using Pearson χ^2^, Fisher exact, and Wilcoxon rank-sum tests as appropriate. One-year survival was compared using Cox proportional hazards regression models, adjusting for age, sex, and CD4 cell count, and Kaplan–Meier survival curves.

## RESULTS

**Figure 1.**
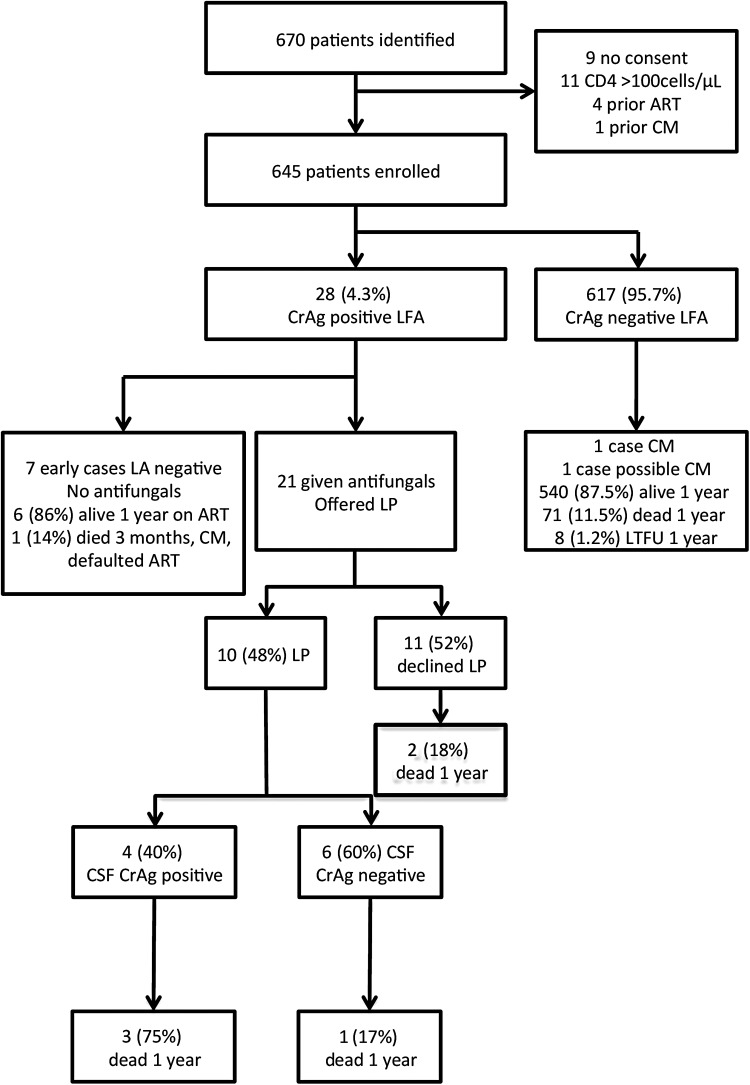
Flow of patients from study inclusion through to discharge. Abbreviations: ART, antiretroviral therapy; CM, cryptococcal meningitis; CrAg, cryptococcal antigen; CSF, cerebrospinal fluid; LA, latex agglutination assay; LFA, lateral flow assay; LP, lumbar puncture; LTFU, lost to follow up.

A total of 670 patients were screened, of whom 645 met eligibility criteria and consented (Figure 1).

### Cryptococcal Antigen Prevalence

Twenty-eight of the 645 patients (4.3%) screened were CrAg positive using the LFA in plasma and serum, and these results were 100% concordant. Seven of these patients (1.1% of those screened) were also positive by LA; all patients with a positive serum LA had a positive LFA. No significant baseline clinical or laboratory differences were identified between CrAg-positive and CrAg-negative patients (Table 1).

**Table 1.** Baseline Patient Characteristics

Variable	All Patients (n = 645)	CrAg Negative (n = 617)	CrAg Positive (n = 28)	*P* Value
Male (%)	302 (47%)	289 (45.7%)	13(46.4%)	.97
Age, y (median [IQR])	36 (31–42)	36 (31–42)	38.5 (32–43.5)	.26
CD4 count, cells/mm^3^ (median [IQR])	55.5 (28–78)	56 (29–78)	49 (14–90)	.48
Hemoglobin, g/L (median [IQR])	10.9 (9–12.3)	10.7 (8.9–12.3)	10.65 (9.35–12.55)	.65
White blood cell count, cells × 10^9^/mm^3^ (median [IQR])	5.07 (3.84–6.82)	5.09 (3.84–6.85)	5.05 (3.97–6.12)	.66
Creatinine, µmol/L (median [IQR])	68 (58–83)	68 (57–83)	66.5 (58–85)	.81
Alanine transaminase, IU/L (median [IQR])	26 (18–41)	26 (18–41)	24 (17–40)	.63

Abbreviations: CrAg, cryptococcal antigen; IQR, interquartile range.

### Cryptococcal Antigenemia in Urine and Whole Blood

Fifty-seven percent (16 of 28 patients) with a positive serum/plasma LFA had a positive urine LFA. An additional 16 serum/plasma LFA-negative patients also tested positive with the urine LFA. These were considered false positives. On repeat testing of the same urine samples after freezing at −80°C, 12 of the 16 false-positive urine samples were LFA negative, leaving 4 unresolved false-positive urine LFA results.

LFA testing of whole blood from 192 patients, including 8 with a positive serum/plasma CrAg, demonstrated 100% concordance between the LFA results from whole blood and serum/plasma.

### Cryptococcal Antigen Titres

Serial dilutions were performed to determine CrAg titers in serum, plasma, and urine in those screening CrAg positive (see Supplementary Figure 1). There was 100% agreement between serum and plasma titers. There was no agreement between visual LFA CrAg grading performed according to manufacturer's instructions (grades 1–5) and antigen titer. Having a positive serum LA test was associated with higher LFA titers in plasma/serum and urine samples. On average, titers were 2 dilutions lower in urine compared with the corresponding plasma or serum samples.

### Proportion of Patients CrAg-Positive by LFA in Blood With Evidence of Meningeal Involvement

Ten of 28 patients with a positive serum/plasma LFA test consented to LP. Forty percent (4 of 10) were CSF CrAg LFA positive (Table 2). Having a positive CSF CrAg was associated with higher serum/plasma LFA titers (see Supplementary Figure). There were no cases with a positive CSF CrAg with a titer lower than 1:160 in the serum/plasma. All 4 CSF CrAg positive cases were also positive by LFA in urine, with titers greater than 1:20. Three of 4 patients with a positive CSF CrAg were positive by LA in serum. In this small sample, headache and fever were not helpful in distinguishing between patients with and without positive CSF CrAg (Table 2). Patients with a positive CSF CrAg usually had other CSF evidence of meningeal involvement, for example, positive India ink or culture results or raised CSF protein (Table 2).

**Table 2.** Comparison of Serum Cryptococcal Antigen (CrAg)–Positive Patients With a Positive Cerebrospinal Fluid (CSF) CrAg, Negative CSF CrAg, or Those Who Declined to Have an Lumbar Puncture

Symptom	CSF CrAg+ (n = 4)	CSF CrAg− (n = 6)	No Lumbar Puncture (n = 18)
Headache	1/4	3/6	2/18
Fever	2/4	1/6	2/18
Confusion	0/4	1/6	1/18
CrAg testing			
Serum latex agglutination assay positive	3/4	1/6	3/18
Urine lateral flow assay positive	4/4	3/6	9/18
CSF parameters			
CSF CrAg titers (n = 3)	Neat, 40, >2560	NA	NA
India ink positive	3/4	0/6	NA
CSF culture positive	2/3^a^	0/6	NA
CSF protein, g/L (n raised [median])	2/4 (0.54)	0/5(0.21)	NA
CSF glucose (n <50% of serum [median])	2/4(2.5)	1/5(3)	NA
CSF white blood cell count (range), cells/mm^3^	0–1	0–2	NA
Blood culture			
Positive for *Cryptococcus neoformans*	2/4	0/6	0/18
Mortality			
10 wk	2/4	1/6	1/18
6 mo	3/4	1/6	3/18
1 y	3/4	1/6	3/18

Abbreviations: CrAg, cryptococcal antigen; CSF, cerebrospinal fluid; NA, not applicable.

^a^ One patient did not have CSF cultured due to laboratory error, hence 2/3 rather than 2/4.

### Treatment of CrAg-Positive Patients and Outcomes

The 4 patients diagnosed with CM on the basis of a positive CSF CrAg were treated with amphotericin B for a median of 14 days, followed by fluconazole. Of these 4 patients, 2 died in the first 10 weeks and 1 died 6 months later. Two patients died prior to starting ART. Time to ART for the other patients was 20 days and 26 days. None of these deaths were directly attributable to CM, although CM-IRIS could not be ruled out in 1 case (see Supplementary Table 1).

Of the remaining 24 serum/plasma CrAg-positive patients, 7 were identified as LFA positive/LA negative prior to LFA approval and were therefore not treated with fluconazole. Six of these patients were started on ART (median time to ART, 3.1 weeks; interquartile range [IQR], 2–9.1). All 6 were alive at 1 year and none developed CM. The remaining LFA CrAg-positive–LA CrAg-negative patient defaulted all follow-up and died of CM 3 months later, having never initiated ART. Seventeen patients treated on the basis of the positive LFA result (4/17 also LA positive), known to have a negative CSF CrAg or who declined LP, were treated with fluconazole. In these patients, ART was started at a median of 2.9 weeks (IQR, 2–4). Two of these patients had died by 10 weeks and 3 had died by 12 months. None of these deaths were attributed to CM (see Supplementary Table 1).

Outcomes were ascertained for all 28 CrAg-positive patients. Mortality was 14.3% (4/28) at 10 weeks and 25% (7/28) at 12 months. Other than the 4 patients with proven CSF antigenemia at baseline, no CrAg-positive patients who were prescribed fluconazole and ART went on to develop CM. No patients with proven CSF antigenemia at baseline developed cryptococcal IRIS, with the possible exception of case 3 (see Supplementary Table 1).

### Treatment of CrAg-Negative Patients and Outcomes

**Figure 2.**
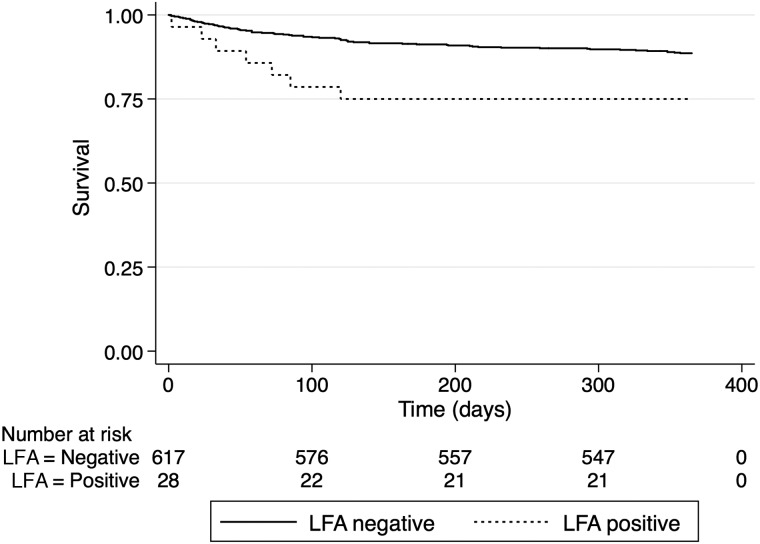
Kaplan–Meyer survival curves showing survival over time in patients screening cryptococcal antigen (CrAg) positive with lateral flow assay (n = 28) compared with those screening CrAg negative (n = 617). Abbreviation: LFA, lateral flow assay.

CrAg-positive patients had a significantly higher risk of death than CrAg-negative patients over the first year on ART (hazard ratio [HR] for death = 2.46; 95% confidence interval [CI], 1.13–5.36; *P* = .023; Figure 2). Of the 617 patients who were CrAg negative on serum/plasma LFA testing, mortality was 5.3% (n = 32) at 10 weeks and 11.5% (n = 71) at 1 year. Eight patients were lost to follow-up, and the remaining 539 were alive at 1 year. This mortality difference remained highly significant after adjustment for age, sex, and CD4 count (adjusted HR = 2.44; 95% CI, 1.12–5.32; *P* = .024). Presumed causes of death in the CrAg-negative patients are shown in Table 3; 23% of deaths were of unknown cause. In none of these unknown cases was cryptococcal disease suspected.

**Table 3.** All-Cause Mortality of Cryptococcal Antigen (CrAg)–Positive and CrAg-Negative Patients

Cause of Death^a^	CrAg Negative	CrAg Positive	Total
Tuberculosis pulmonary/tuberculosis undefined	27	0	27
Tuberculosis extra-pulmonary	5	1	6
Tuberculosis IRIS	3	0	3
Sepsis, all cause	13	2	15
Renal failure	1	1	2
Measles	1	0	1
Colonic cancer, metastatic	0	1	1
Cytomegalovirus encephalitis/hepatitis	1	0	1
Toxoplasmosis	1	0	1
Varicella Zoster Virus meningitis	1	0	1
Cryptococcal meningitis	1	1	2
Unknown	17	1	18
Total	71	7	78

Abbreviations: CrAg, cryptococcal antigen; IRIS, immune reconstitution inflammatory syndrome.

^a^ Cause of death was ascertained where possible using electronic databases, death certificate data, and patient hospital notes review.

One definite case and one possible case of cryptococcal disease were identified in patients who screened CrAg negative: 1 patient started ART 10 days after screening and was virally suppressed at week 28 with a good CD4 cell recovery (40 cells/µL at baseline, 140 cells/µL at week 28). The patient developed confirmed disseminated tuberculosis at week 29 and started anti-tuberculosis treatment. He was admitted to the hospital at week 31 with seizures and a stroke. CSF was reported as protein >5 g/L, glucose 0.3 mmol/L, lymphocytes 57 cells/µL, CrAg positive, and India ink and culture negative. CSF was not cultured for *Mycobacterium tuberculosis*, and no peripheral CrAg was done. Amphotericin B followed by fluconazole were added to anti-tuberculosis therapy, and the patient made a good recovery. In retrospect, it was unclear whether this was cryptococcal or tuberculous meningitis with false-positive CSF CrAg. A second CrAg-negative patient was admitted to the hospital on day 364 of the study with CrAg and *Cryptococcus neoformans* culture-positive CSF. That patient had had poor adherence to ART (week 48 viral load 268 000 copies/mL, CD4 207 cells/µL) and had defaulted clinic follow-up.

## DISCUSSION

In this prospective study of patients with low CD4 counts initiating ART in South Africa, CrAg screening and preemptive fluconazole treatment of CrAg-positive patients was associated with markedly fewer cases of and deaths from CM compared with historic unscreened cohorts. In a historic control cohort from one of the study clinics, 28% of LA CrAg-positive patients who did not receive preemptive antifungal therapy went on to develop proven CM [7]. Our findings in this study suggest that preemptive antifungal therapy and timely ART initiation prevents CrAg-positive patients without meningeal involvement from developing CM or CM-IRIS in the first year of ART. Our results also confirm findings from earlier studies [13, 21] that showed that the large majority of patients screen CrAg negative (>95% in this cohort) and are not at significant risk of clinical cryptococcal disease if ART is started promptly after screening and if patients adhere. Importantly, screening did not lead to delays in ART initiation or decreased retention in care.

However, although cryptococcal disease was thought to be related to just 2 of 78 (2.6%) deaths in the first year of ART (both of these cases in patients who were nonadherent to ART) compared with up to 20% of deaths in historic, unscreened cohorts [6–8, 22], the mortality of CrAg-positive patients remained significantly higher than that of CrAg-negative patients, even after adjustment for CD4 count and despite preemptive antifungal therapy. This is consistent with data from other recently published prospective CrAg screening studies [23–25]. One possible explanation is that the CrAg-positive patients were dying of undiagnosed cryptococcal disease; however, all patients were closely followed up by the study team and alternative causes of death were ascertained (see Supplementary Table 1). Another explanation is that the CrAg-positive patients are also at increased risk of dying from other AIDS-related conditions [26, 27]. Cryptococcal antigen itself has significant immunosuppressive effects [28], providing one possible explanation for these observations. Another is that CrAg positivity is a marker of profoundly impaired immunity (not adequately reflected by the CD4 count alone). Differences in host genetics may determine why some patients, despite presumed widespread exposure to *Cryptococcus*, develop disseminated infection and others do not. Such differences may also be reflected in increased susceptibility to additional opportunistic infections and AIDS-related malignancies.

The cryptococcal LFA yielded 4-fold more positive samples compared with the LA, confirming its significantly higher sensitivity [29, 30]. It was also shown to perform well on whole blood samples, meaning true point-of-care screening should be possible using finger prick blood samples [31]. Unfortunately, in asymptomatic or minimally symptomatic patients, the sensitivity of the LFA using urine was low (57% relative to serum/plasma), and more problematically, there were as many false-positive as true-positive results, yielding a 50% positive predictive value in this context. This confirms other recent reports that showed low sensitivity of urine LFA when used to screen patients initiating ART and false-positive results [32]. The LFA is not approved for use with urine and, on the basis of these findings, cannot be recommended for screening asymptomatic individuals. Positive urine samples must be confirmed by testing another approved specimen type.

Interestingly, the prevalence of asymptomatic antigenemia was lower than predicted (4.3% with sensitive LFA and only 1.1% with the LA). In comparison, the antigen prevalence was 6% using the LA in a cohort with CD4 cell counts ≤100 cells/µL and no prior cryptococcal meningitis drawn from the same patient population between 2002 and 2005 [13]. Similar results are seen in current screening data from Gauteng who detected 4% CrAg prevalence using the LA in 2009–2010 and 4% more recently using the LFA [33, 34]. Reasons for this reduction in CrAg prevalence in the patient group with a CD4 cell count ≤100 cells/µL are not clear. The risk of reactivation of or new infection with *C. neoformans* is likely to be related to the duration of severe immunosuppression; current patients with a CD4 cell count <100 cells/µL in South Africa may not have been at this level of immunosuppression for as long as patients in prior years due to improvements in ART provision and access [23]. It will be important to see if reductions in antigen prevalence occur in other centers and countries over time, as the cost-effectiveness of CrAg screening interventions are related to CrAg prevalence in the screened population [16].

Meningeal involvement was present in 40% of patients screening CrAg positive who consented to LP, even in the absence of marked symptoms. However, it would be difficult to routinely offer LPs to all patients who screen CrAg positive in African ART programs. Of the 11 CrAg-positive patients who declined LP but took fluconazole, 4 had a serum titer ≥1:160 and none developed CM, providing preliminary evidence that high-dose fluconazole plus ART is sufficient to prevent the development of clinical CM in the majority of cases. Whether or not LPs are required to guide management in asymptomatic CrAg-positive patients remains to be determined, but meningeal involvement in this study was associated with higher antigen titers, raising the possibility that lumbar punctures and/or more aggressive antifungal therapy could be targeted to those with higher antigen titers.

Our study provides important new prospective data to inform CrAg screening interventions in patients with low CD4 cell counts entering ART programs. However, optimal strategies for implementing screening still need to be defined. The very high mortality in CrAg-positive patients despite antifungal therapy suggests that CrAg screening may be best implemented as part of a combined opportunistic infection (OI) screening and intervention package for patients in this high-risk group. Further work is needed to better understand the persisting high mortality and how best to incorporate screening for CM and other OIs into routine care pathways.

## Supplementary Data

Supplementary materials are available at http://cid.oxfordjournals.org. Consisting of data provided by the author to benefit the reader, the posted materials are not copyedited and are the sole responsibility of the author, so questions or comments should be addressed to the author.

Supplementary Data
