# Supplementary material for: Cryptococcal Antigen Screening in Patients Initiating ART in South Africa: A Prospective Cohort Study
Source: Clin Infect Dis. 2015 Nov 12;62(5):581–7. doi: 10.1093/cid/civ936 (PMC4741358; doi:10.1093/cid/civ936)
Supplement: Supplementary Data [file supp_62_5_581__index.html]

Cryptococcal Antigen Screening in Patients Initiating ART In South Africa: A Prospective Cohort Study — Cryptococcal Antigen Screening in Patients Initiating ART in South Africa: A Prospective Cohort Study — Cryptococcal Antigen Screening in Patients Initiating ART in South Africa: A Prospective Cohort Study — Supplementary Data 

# Cryptococcal Antigen Screening in Patients Initiating ART in South Africa: A Prospective Cohort Study

## Supplementary Data

Supplementary Data

- Supplementary Figure - pdf file
- Supplementary Table - pdf file
