# Supplementary material for: Cryptococcal Antigen Screening in Patients Initiating ART in South Africa: A Prospective Cohort Study
Source: Clin Infect Dis. 2015 Nov 12;62(5):581–7. doi: 10.1093/cid/civ936 (PMC4741358; doi:10.1093/cid/civ936)

Supplemental Figure

A. LFA titres in serum/plasma in CrAg positive patients and relationship to LA positivity. [top] **B.** LFA titres in urine in serum CrAg positive patients and relationship to LA positivity [bottom]

A

B

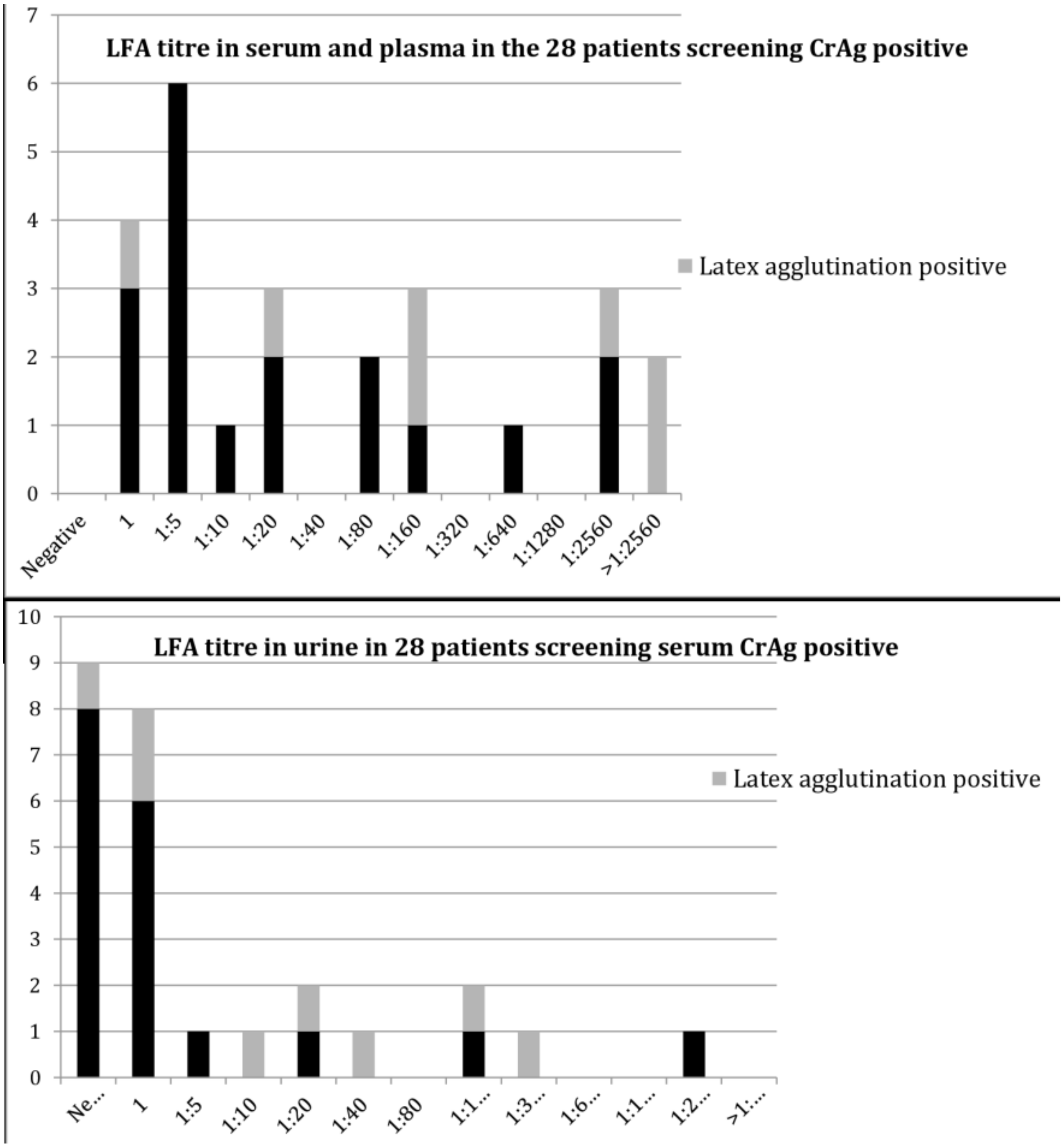

C. LFA titres in the 10 serum CrAg positive patients agreeing to an LP with corresponding CSF LFA and serum / plasma LA results

C

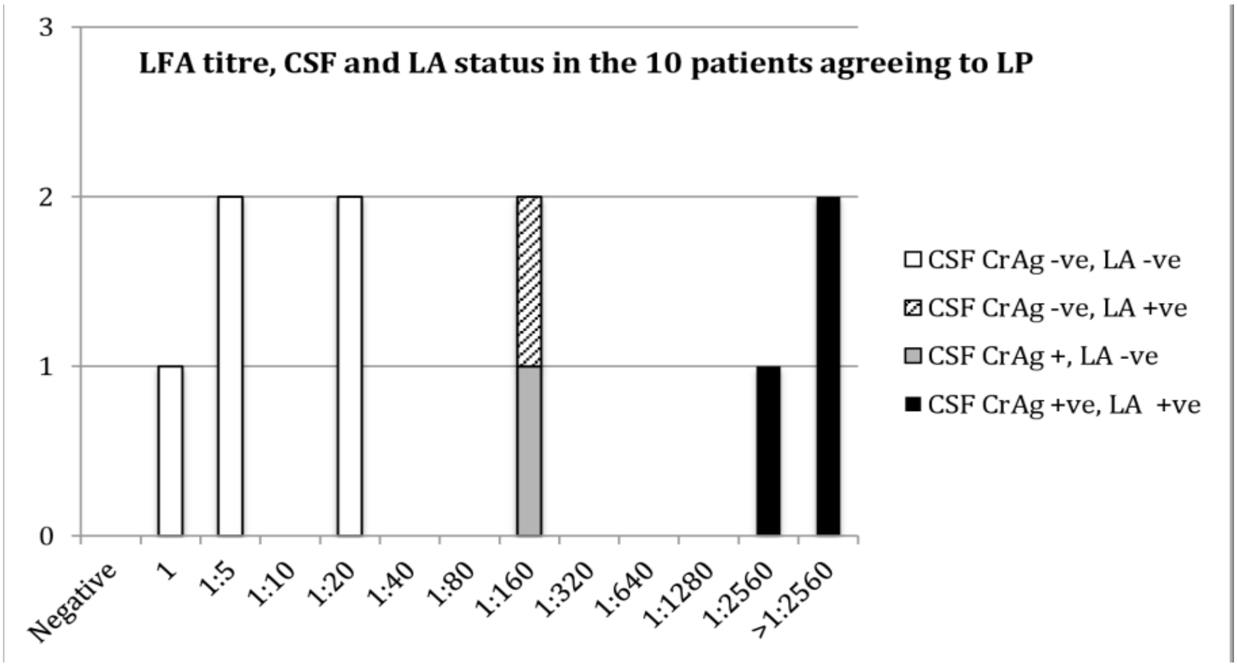

Supplement: Supplementary Data [file supp_civ936_civ936supp_fig.pdf]
