# Supplementary material for: Cryptococcal Antigen Screening in Patients Initiating ART in South Africa: A Prospective Cohort Study
Source: Clin Infect Dis. 2015 Nov 12;62(5):581–7. doi: 10.1093/cid/civ936 (PMC4741358; doi:10.1093/cid/civ936)
Supplement: Supplementary Data [file supp_civ936_civ936supp_table.pdf]

Supplemental Table. Details of deaths in patients screening positive for CrAg

	Cause of Death	Day of Death	CFS CrAg Result	Antifungal Therapy	Narrative
**1**	Bacterial infection: MRSA	85	Positive	AmB induction	Widespread erythroderma, lung abscess and bacteremia. Self-discharged from hospital prior to completion of antimicrobials
**2**	Bacterial infection: Nosocomial pneumonia	21	Positive	AmB induction	Admitted with disseminated TB, renal and hepatic impairment. Improvement of CM and TB. Developed nosocomial pneumonia and died despite appropriate antibiotics
**3**	Unknown/possible CM IRIS	33	Positive	AmB induction	Uncomplicated CM treated with 2 weeks IV AmB. 1week post discharge developed gastroenteritis. Manage at home with ORS and fully recovered. Died at home on day 12 ART, no preceding headache or illness
**4**	Right upper lobe pneumonia and acute renal failure	27	Negative	Fluconazole	Developed ATN secondary to sepsis and tenofovir.
**5**	Metastatic adenocarcinoma of the colon	112	Not Done	Fluconazole	Multiple bone and liver metastases
**6**	Disseminated TB	2	Not Done	Fluconazole	Admitted on day of screening with smear positive rifampicin sensitive disseminated TB.
**7**	Cryptococcal Meningitis	53	Not done	Nil	Screened CrAg positive with LFA prior to FDA approval and negative with the LA. Defaulted clinic and did not start ART. Admitted with and died of CM.

Supplemental Table. Details of deaths in patients screening positive for CrAg
